# Patient and public involvement to inform priorities and practice for research using existing healthcare data for children’s and young people’s cancers

**DOI:** 10.1186/s40900-023-00485-8

**Published:** 2023-08-29

**Authors:** Nicola F. Hughes, Lorna A. Fern, Angela Polanco, Chris Carrigan, Richard G. Feltbower, Ashley Gamble, Emily Connearn, Angela Lopez, Ellen Bisci, Kathy Pritchard-Jones

**Affiliations:** 1 Leeds Institute of Medical Research, School of Medicine, University of Leeds, Leeds, UK; 2 Cancer Clinical Trials Unit, University College London Hospitals, London, UK; 3 Public Contributor, CCLG, Leicester, UK; 4 DATA-CAN, Leeds Institute for Data Analytics, School of Medicine, University of Leeds, Leeds, UK; 5 Leeds Institute for Data Analytics, School of Medicine, University of Leeds, Leeds, UK; 6 Childhood Cancer and Leukaemia Group (CCLG), Leicester, UK; 7 UCL Great Ormond Street Institute of Child Health, University College London, London, UK; 8 Young Person’s Representative, Leicester, UK

**Keywords:** Children and young people with cancer, Healthcare data, Participatory patient and public involvement methods, Outcomes research

## Abstract

**Background:**

In the United Kingdom, healthcare data is collected on all patients receiving National Health Service (NHS) care, including children and young people (CYP) with cancer. This data is used to inform service delivery, and with special permissions used for research. The use of routinely collected health data in research is an advancing field with huge potential benefit, particularly in CYP with cancer where case numbers are small and the impact across the life course can be significant.

Patient and public involvement (PPI) exercise aims:

- Identify current barriers to trust relating to the use of healthcare data for research.
- Determine ways to increase public and patient confidence in the use of healthcare data in research.
- Define areas of research importance to CYP and their carers using healthcare data.

**Methods:**

Young people currently aged between 16 and 25 years who had a cancer diagnosis before the age of 20 years and carers of a young person with cancer were invited to take part via social media and existing networks of service users. Data was collected during two interactive online workshops totalling 5 h and comprising of presentations from health data experts, case-studies and group discussions. With participant consent the workshops were recorded, transcribed verbatim and analysed using thematic analysis.

**Results:**

Ten young people and six carers attended workshop one. Four young people and four carers returned for workshop two. Lack of awareness of how data is used, and negative media reporting were seen as the main causes of mistrust. Better communication and education on how data is used were felt to be important to improving public confidence. Participants want the ability to have control over their own data use. Late effects, social and education outcomes and research on rare tumours were described as key research priorities for data use.

**Conclusions:**

In order to improve public and patient trust in our use of data for research, we need to improve communication about how data is used and the benefits that arise.

**Supplementary Information:**

The online version contains supplementary material available at 10.1186/s40900-023-00485-8.

## Abstract (plain language summary)

Everyday data is collected on all patients treated within the National Health Service, including children and young people with cancer (CYP). This data is used routinely to improve how services are run and with special permissions, can also be used for research. Negative reporting in the media about this use of data can lead to mistrust and some people choosing not to share their data. This can reduce the quality and accuracy of research looking at rare diseases or populations with small numbers. In addition, many barriers exist to researchers when trying to access this data such as laws around data sharing, making it difficult and sometimes impossible to carry out such research. We invited CYP and carers to two workshops to:

- Learn about how healthcar e data is used for research.
- Consider ways to increase public and patient confidence in this use of healthcare data.
- Describe areas of research importance to CYP and their carers using healthcare data.

Ten young people and six carers attended the first workshop. Four young people and four carers returned for workshop two. Workshops consisted of interactive presentations, case studies and group discussions. Overall participants felt that lack of awareness and negative media reporting led to mistrust in data use for research. It was believed that greater education about how the data is used, including positive examples of the benefits of the research, was needed to improve public confidence. Key research priorities for data use included late-effects, social and educational outcomes and rare tumours.

**Supplementary Information:**

The online version contains supplementary material available at 10.1186/s40900-023-00485-8.

## Background

Large amounts of data are collected every day in the routine care of patients with or surviving from cancer. This includes data collected in primary and secondary National Health Service (NHS) care, educational and social settings and covers a wide range of information including details relating to the patient, (e.g. age at diagnosis and ethnicity), their cancer (e.g. size and spread at diagnosis) and information about any health conditions occurring after treatment. The use of routine healthcare data to improve patient outcomes has been gathering momentum over the past few years, though its use is restricted by governance frameworks as outlined in the recent Goldacre report [1]. The COVID-19 pandemic demonstrated the power and efficacy of timely data collection, access, linkage, analysis and reporting. In the United Kingdom (UK) the government intends to continue to embrace the power of data as outlined in their policy “Data saves lives: reshaping health and social care with data [2].” Cancer Research UK also plan to utilise the enormous potential held in data driven research with the release of their research strategy “Unleashing the power of data to beat cancer” [3].

Cancers occurring in children and young people (CYP) are rare, but despite this remain a leading cause of death in these age groups. Nationally, approximately 1645 new cases are diagnosed annually in 0–14 year olds and approximately 2110 in 15–24 year olds [4]. CYP also experience rarer cancers [5], for example, lymphomas, brain cancers, sarcomas and germ cell tumours, compared to older adults where breast, colorectal, prostate and lung dominate [6]. Therefore, data sharing is crucial to improve our knowledge of CYP cancers and to strengthen the research being carried out. This includes sharing information safely and securely between institutions in the UK and also internationally.

In England, routine healthcare data is collected for research purposes without patient consent under Section. 251 of the NHS Act 2006. Individuals do however have the right to opt out of their health records being shared for purposes other than direct care. In June 2021, there was widespread reporting in the media of the NHS “data grab” from General Practitioner (GP) records, a scheme in which GP health data for patients in England would become more readily available for research and health service planning [7]. This resulted in a significant increase in individuals opting out of data sharing with numbers almost doubling from 1,652,082 to 3,220,803 over a three-month period [8]. For rare diseases such as CYP cancers even small numbers of individuals opting out can have a significantly disproportionate effect on the generalisability of research results. To minimise the numbers of people opting out, the population must trust those using the data to do so safely in order for healthcare data to reach its potential.

As part of the British Science Associations (BSA) Future Forum 2020, 14 young people aged 14–18 years were asked about the use of medical data. More than half, (61%) felt they did not know much or anything about how medical data gets used. With 70% reporting they trusted the NHS to process their data, this fell to 31% for universities/ academic institutions, 23% for Government and 18% for pharmaceutical companies [9]. Although this report represents the views of a small sample, it suggests a lack of knowledge about data collection amongst young people and varying levels of trust regarding the use of medical data. We invited CYP and their carers to a patient and public involvement (PPI) workshop to learn how their healthcare data is used and to:

- Identify current barriers to trust regarding the use of healthcare data for research.
- Determine ways to increase public and patient confidence in the use of healthcare data in research.
- Define areas of research importance to CYP and their carers using healthcare data.

## Methods

Two workshops were carried out in January and April 2022 each lasting 2.5 h. Participants were recruited to the initial workshop following advertisement via DATA-CAN, use MY data, the Children’s Cancer and Leukaemia Group (CCLG), the Teenage Cancer Trust (TCT), Candlelighter’s Trust and the research teams’ social media accounts. Adverts were circulated two weeks prior to workshop 1. Young people currently aged between 16 and 25 years who had a cancer diagnosis before the age of 20 years, and carers of a young person diagnosed with cancer before the age of 20 years were invited to take part. A £50 incentive was offered in return for taking part in each workshop. We selected an upper age eligibility criteria of 20 years as this was in keeping with the age eligibility criteria of the International Benchmarking of childhood cancer survival by stage (BENCHISTA) project [10]. Participants from workshop 1 were invited back to workshop 2. The workshops were facilitated by experienced qualitative researchers AP (CCLG) and LF (UCLH). The research team consisted of KPJ (University College London (UCL) and DATA-CAN), CC (DATA-CAN), RF and NH (Yorkshire Specialist Register of Cancer in Children and Young People (YSRCCYP) and University of Leeds), EC (DATA-CAN), AL (UCL) and AG (CCLG).

### Workshop format

Due to COVID-19 restrictions the workshops were held online via a secure Zoom account. The workshops were recorded, transcribed verbatim and stored in line with the University of Leeds data security procedures. All participants provided informed consent prior to taking part using an online consent form. The workshop schedules and case studies are available in Additional files 1 and 2.

### Workshop 1

Workshop 1 started with an interactive presentation (CC) covering; how patient data is generated in the healthcare system, what data this includes and the different levels of identifiability. The collection, storage, and use of data by cancer registries and clinical trials was described along with consent and the data opt out policy.

Participants were then divided randomly into two break-out rooms to discuss two different case studies relating to a young person diagnosed with cancer. The groups were asked to consider what data might be collected and when and who might have access to this data.

RF presented research carried out by the YSRCCYP, which had used linked NHS datasets, to improve survival rates for childhood and young peoples’ cancers. NH presented the research cycle, and how data is used and shared throughout.

Participants were invited to ask any questions and feedback their views following each presentation. The final discussion focused on asking the participants to consider ways in which awareness of data use for research purposes could be improved.

### Workshop 2

In workshop 2 examples of anonymised primary care, hospital and cancer registration records were used to show participants what data records look like (CC). These examples were then used to explain the difference between data linkage and data sharing, covering aspects including data minimisation and data security measures (CC). A presentation of the BENCHISTA [10] project (KPJ/AL) provided an example of research which requires international data sharing. This facilitated a discussion regarding the legislative barriers faced by such projects and gave participants the opportunity to feedback their thoughts.

A presentation (RF and NH) was given covering the use of social outcome measures in cancer research, why it is important, and barriers faced in accessing the data. A discussion surrounding the use of these data sources followed (LF).

Prior to the workshop participants had been provided with three newspaper articles reporting on use of patient data. Participants were invited (CC) to discuss their thoughts on the articles in relation to data sharing and how such articles may be perceived by the public. The workshop ended with a discussion of key learning points from the workshops and areas for future work. (AP).

### Analysis

The workshop transcripts were analysed using thematic analysis [11]. This involved familiarisation with the data, generation of codes and examining, reviewing, and defining themes. The initial generation of codes was carried out and agreed by NH and LF, draft themes were then reviewed and finalised by the wider team. Subthemes were then devised by NH and agreed by the wider team. The supporting quotes presented in the results section were selected as they were felt to succinctly represent identified themes and are presented using intelligent verbatim. YP denotes a quote from a young person and C from a carer.

Our findings are reported in line with the GRIPP2 checklist (Additional file 3).

## Results

Ten young people currently aged 16–25 years and diagnosed with cancer under the age of 20 years, and six carers of young people with cancer responded to the advert and all took part in workshop 1. Four young people and four carers took part in workshop 2. The participants did not know each other prior to the workshops. Participant characteristics are shown in Table 1.

**Table 1:** The characteristics of workshop one participants

Characteristic	Workshop 1 n (%)			Workshop 2 n (%)		
	Total (n = 16)	YP (n = 10)	C (n = 6)	Total (n = 8)	YP (n = 4)	C (n = 4)
Sex						
Female	13 (81)	8 (80)	5 (83.3)	5 (62.5)	2 (50)	3 (75)
Male	3 (19)	2 (20)	1 (16.7)	3 (37.5)	2 (50)	1 (25)
Ethnicity						
White	12 (75)	8 (80)	4 (66.7)	5 (62.5)	2 (50)	3 (75)
Asian/mixed	4 (25)	2 (20)	2 (33.3)	3 (37.5)	2 (50)	1 (25)
Current age (years)						
16–25	10 (60)	10 (100)	0 (0)	4 (50)	4 (100)	0
Over 25	6 (40)	0 (0)	6 (100)	4 (50)	0	4 (100)
Cancer type of CYP*						
Solid tumour	4 (40)	4 (40)	–	2 (50)	2 (50)	–
Haematological	6 (60)	6 (60)	–	2 (50)	2 (50)	–
Current country of residence						
England	14 (87.5)	9 (90)	5 (83.3)	7 (87.5)	3 (75)	3 (75)
Scotland	2 (12.5)	1 (10)	1 (16.7)	1 (12.5)	1 (25)	1 (25)

*This data is only reported for young people as understandably some bereaved parents did not want to share this information

Three main themes were identified; existing barriers to trust in healthcare data use for research, ways to improve public and patient confidence and research priorities for data use. These are discussed in turn with appropriate quotes form participants.

### Existing barriers to trust in healthcare data use for research

This theme encompassed the sources of mistrust and how a lack of awareness about healthcare data use presents barriers to trust.

#### Lack of awareness

Participants were generally unaware of the level of healthcare data that is collected about them, or that this occurs during primary care and secondary care appointments and during follow up. This was particularly true for young people who had their parents advocating for them during treatment as children. There was even less awareness around the collection of social and educational outcomes.

“I didn’t really have any idea of all the data collected. Especially when I was going through treatment … I just didn’t have any part in that area … I just got my Mum to sort everything. So it’s quite eye opening.” YP-6.

“Same for me. You just don't really think about it and it's so in depth as well.” YP-3.

Whilst some participants knew they were consenting for a procedure such as the collection of a tissue sample, they were unaware that this would produce data which would be collected and used.

“Sometimes it's not obvious data gets produced from something. My child had a tissue sample taken… there would have been digital data produced and that's quite difficult to imagine … that's not something I'd visualised before.” C-1.

“I certainly wasn't ever told about what my data was going to be used for. I suppose before surgeries, and all that sort of stuff you always got told what was going to happen but certainly not what the results were going to be used for.” YP-5.

#### Sources of mistrust

Negative portrayal of data use by the media, such as stories about patient health care records being sold to private companies were seen as lowering public confidence in data sharing. Experiences of receiving “spam” advertising via email or text were also described as lowering confidence and demonstrated a lack of awareness of different types of data sharing, for example the difference between data sharing for marketing purposes and data sharing for research.

“How the media portrays it (data use)… has negative impacts and it does put you off data sharing.” YP-2.

“I think part of the problem is that when we talk about data and research, a lot of us start thinking about how we get adverts for things because we've clicked on something.” C-2.

### Ways to increase public and patient confidence

This included providing more information about data collection and use and giving young people more responsibility for their own data.

#### More information about data use in research

Participants felt that having more information available about how data is used for research and safety measures that exist, for example data security and anonymisation of data, would improve public confidence.

“I think it's quite important to highlight that the data is very well organised and very well protected”. YP-1.

“You just have to get it out there somehow like, get it on the internet and things. I feel like people are worried about having really identifiable information about themselves, distributed to loads of different companies. To kind of reassure people that really most of this data is not identifiable …no one can connect it to you… it would actually be hugely reassuring.” YP-7.

Dissemination of positive outcomes from research using healthcare data were seen as an important way of improving awareness of, and therefore trust in, data use. Additionally, embedding data use in the current curriculum was described as a way to raise awareness of collection for research purposes and of security measures in place to protect the data and anonymity.

“I do wonder whether, case studies of positive uses of data and research need to be a little bit more embedded in school curriculum, so that we can develop skills as a society to differentiate.” C-2.

“There are multiple positive impacts that I feel aren't shared as loudly and it's just the way it's presented to the public. I think it’s important to try and show the benefits that can be achieved.” YP-2.

“How the information is presented, that is key here. If it’s explained to you clearly and that it's in the best interest of the public, and yourself … there won’t be barriers.” C-6

#### Ability to take responsibility for own data

Some of our participants were diagnosed as children, meaning their parents or carers advocated for them in decisions regarding healthcare data sharing. These individuals described a transition of responsibility for their data. Initially when diagnosed they were happy for their parents to take charge but now with increased age and distance from treatment, they want to be able to make those decisions for themselves.

“There's an assumption that we can't have those conversations (about data use)… with young people.” C-4.

“Being so young, when I was diagnosed, my parents made most of those decisions for me about data and so I didn't really comprehend that anything was going to be shared… as an adult now, I'd like to feel like I had control of the data or at least continued the consent to use it.” YP-1.

There was a general consensus that there is a need for increased awareness regarding the use of healthcare data to enable individuals to take responsibility for their own data. However, there was uncertainty around the best time to provide this information. Most participants felt that providing this information at the time of diagnosis was inappropriate. There was concern that for some individuals unexpectedly receiving this information sometime after treatment may be triggering of emotions felt during treatment.

“I'm in two minds about it. On the one hand, I feel if I just received a random letter in the post saying here's how your data has been used in the past … years since your diagnosis, I'd feel obliged to read it, but knowing that, that could very easily trigger my brain. Almost blissful ignorance is better, like I gave you my data that's yours now. I don't particularly want to think about that time I had the biopsy or that time that I had that treatment. Whereas with this (workshop), I was invited to do this, I'm mentally prepared for it. That's totally cool. But if that information was then sprung on me, I don't think I'd be ready for that.” YP-1.

“When I was first diagnosed, I think if you'd sat me down and said all your data is going to be used for X Y, Z, I probably wouldn't have cared less. My whole attitude towards the entire thing was, let's just get the treatment. Let's get it done. But certainly now and certainly after I've had all my surgeries, all the chemo and all that sort of stuff, it would be interesting to go back and say, oh, yeah, your data was used for this, this this. So I think maybe at the end of the treatment.” YP-7.

### Research priorities for data use

Participants described late effects, social and educational outcomes and rare cancers as areas of research importance.

#### Late effects

Research into the late effects of cancer and the treatment was important to participants. Some gave examples where they felt they had directly benefitted from such research.

“To pick up on the point about gathering data after treatment finishes. I've always been really grateful about knowing about the long-term side effects that my child might have. People used to say, once you finish their treatment within six months they'll feel a lot better, they'll get their energy back, be able to play sport just like a normal child. And that hasn't happened. Because people have allowed their data to be used, because of the research that's happened, we've been able to see that actually, they might have long term side effects and their mobility might continue to be affected. We might not have known that if people hadn't done the research into long term side effects.” C-2.

“I think it’s really important that especially information on late effects is available. When I was diagnosed I was 13, fertility was just not mentioned to me. That was something that I had to go out and seek for myself. So if it weren’t for that information and that data being out there…, I would never have known that I could go and ask somebody about my fertility and … seek help on that aspect.” YP 1.

#### Social and educational outcomes

Participants felt that research using social outcomes data would be valuable, particularly as social outcomes can impact children and young people more than older adults due to the amount of time lost in education or work.

“It's really important, I think, often, the social outcomes side can be really neglected, with people obviously focused on health. But that (social outcomes) can have a massive impact on people's lives in other ways.” YP-7.

“I'm someone who struggled with education and employment… I think it's really important. I think it's something that’s not really looked into enough. So yeah, I'm all for it”. YP-4.

Despite the support for research around social outcomes it was, however, acknowledged that this type of data could be seen to be more sensitive than healthcare data.

“I think it is a more personal area as you don't really have a choice on your cancer, like what your diagnosis is, but you have a choice about how you act with it afterwards. I wouldn't mind giving my data, I feel like other people would feel more judged based on the data they're providing.” YP-4.

“I think it is more sensitive than some of the health data just because I think for some people, it seems more personal than scientific stuff that feels out of your control.” YP-7.

#### Rare tumours and outcomes

The importance of data sharing, including internationally, in young onset cancers, particularly where certain tumours and outcomes can be rare, was described by participants. They could see the value data sharing could have for rare tumours and felt it was important their data was shared for these reasons.

“If someone else finds themself in the same situation as you, it can help massively with research and helping outcomes and treatment for children and young people. We've all in a way got a responsibility to do our bit.” YP-9.

“I think it's very important, not just for rare diagnoses, but also for diagnoses that are quite common in a certain particular group but other people get them too. My diagnosis, it's very common in elderly. I got it when I was very, very young.” YP-2.

“I was just thinking that, if you have got something like an anomaly … the more data you have, there might be more anomalies that then spark ideas for new research. Those sort of pathways are kind of shut off without sharing.” YP-1.

#### Implementation of findings

The key findings from the workshops are summarised in Table 2 alongside actions taken by our research teams to improve public and patient trust in our research and inform our research strategy. These actions include continuing to work with workshop participants, embedding the patient and public voice into our research.

**Table 2:** Key findings from the workshops and actions taken by the research teams

Key learning point	Action taken
*Increasing public and patient trust*	
Improved awareness of how the data is used including positive outcomes of data use	Public engagement events e.g. Be Curious at University of Leeds Conference presentations including parent and young persons’ and carer representatives Easily accessible websites written in plain English Patient information resources at sites of data collection e.g. cancer outpatient clinics, teenage cancer wards Incorporation of infographics showing research outputs into posters Newsletters Twitter
Enable young people to take responsibility of own data	Having information about our data use available for those who want to learn more Continuing to provide the option to opt out of data sharing and stating this on information resources
*Research priorities for CYP using healthcare data*	
Late effects	Successful in a Teenage and Young Adult Cancer (TYAC) grant to look at the risk of kidney injury in Teenage and Young Adult cancer patients using healthcare data. The research advisory group for this project includes three participants from these workshops PhD project looking at cardiometabolic late effects Collection of Patient Reported Outcome Measures (PROMs) which patients can view and use to make informed treatment decisions
Social and educational outcomes	YSRCCYP continue to pursue these datasets as part of ongoing research objectives
Data sharing in rare tumours	BENCHISTA has successfully gained approval for international data sharing through population-based cancer registries to compare tumour stage at diagnosis of childhood cancers

## Discussion

We report on the first consultation exercise between CYP with cancer, their carers and researchers on the use of their healthcare data for cancer research purposes. Participants reflected a range of cancer types, ages and experiences. Our results reflect the findings of the BSA future forum [9] that there is a lack of knowledge about how healthcare data is used.

Participants were clear they wanted the opportunity to have control over their healthcare data. This is in keeping with healthcare policy “no decision about me, without me” [12] where adolescent and young adult cancer patients have demonstrated that they want to be involved in decisions about their care [13, 14]. Currently individuals aged 13 years and over are able to set a national data opt-out [15]. It is therefore important that we provide young people with the information required to make an informed decision. This needs to be communicated in a balanced way to avoid negative portrayal often provided through the media. For those diagnosed as children, where decisions are generally driven by parents or legal guardians, additional support and information needs to be provided to those transitioning to adolescence from paediatric services, enabling them to start to take on control of their own decisions around healthcare and social data sharing.

The most appropriate time along the cancer pathway to inform CYPs and their families about routine data collection is unclear and is likely to differ between patients and circumstances. Participants pointed out patients newly diagnosed with cancer are likely to be in a high state of anxiety and unable to comprehend all that is being said to them. However, participants felt that an unexpected notification about how their cancer data is being used after the event may be triggering of negative emotions felt during the time of diagnosis and treatment. This is an area that needs sensitive consideration and for which we are receiving valuable input from CYP and their carers along with specialist healthcare professionals to find the optimal solution.

Whilst we have taken action to improve communication about our research, we acknowledge that this is only part of the picture. As suggested in our workshop, building education surrounding data use into the school curriculum may be one possible way forward. Over time this would help to normalise the use of healthcare data for research purposes and build trust within the general public. As researchers we must continue to be transparent in our use of the data and ensure that appropriate resources are there for people who want to find out more. This includes promotion of the good achieved through research using healthcare related data.

Participants in our workshop were supportive of data sharing in CYP cancers. This may reflect the known altruistic behaviour demonstrated by cancer patients and their families [16, 17]. Research into social and educational outcomes was seen as an area of importance by participants. Whilst progress is being made linking education data [18, 19], linkage of employment data from the Department for Work and Pensions is more difficult. The recently published top ten priorities for children’s cancer by the James Lind Alliance include improving long-term outcomes for survivors, adding further support to the importance of this area of research [20]. Internationally, the introduction of European Union General Data Protection Regulation (GDPR) has exacerbated difficulties in international sharing of health and research data, impacting CYP cancer research [21]. Organisations are calling for a harmonised interpretation of the regulations. We need to harness the patient voice, as heard in our workshops, to help break down these legislative barriers both within and outside of the UK.

### Strengths and limitations

A strength of our study is that it was nationally accessible enabling patients from across England and Scotland to participate. The online setting allowed individuals to attend who normally may have been unable to for health reasons or due to educational or work commitments. Participants were able to attend both workshops, enabling them to expand their own knowledge through our highly experienced multidisciplinary research team. We have invited all participants to continue working with us and a number have taken this opportunity, which has benefited our ongoing research (Table 2).

Despite the strengths of our workshop, we acknowledge there are limitations. The participants were a self-selecting cohort and therefore may not reflect the views of all young onset cancer survivors and carers. There was under representation of males which is common in patient and public consultations. A number of strategies have been attempted to increase the number of males participating in PPI including targeting male specific charities and targeted social media campaigns, neither of which have been successful [22]. We must continue to consider ways of increasing the accessibility and attractiveness of our PPI exercises in a bid to include underrepresented groups such as males. One tested method is for the team to attend existing groups where males attend such as testicular cancer support groups [23]. However, our workshops were held when most NHS institutions had restrictions on face-to-face meetings. We were unable to determine the true uptake of the workshops due to the snowballing method of recruitment via social media. Both recruitment for the workshops and the workshops themselves were carried out online. This excludes those without access, who may have different opinions. While online workshop formats support national representation of participants, there are some groups for which online may present barriers. For example, those who are unable to operate the technical aspects of participating online although this is likely to be less of an issue with young people. Young people however may not have unlimited data mobile phone contracts or a private space to connect to Wi-Fi. To overcome this issue, we offered to reimburse any participants who had to purchase additional data to participate. As with many PPI activities, educational status and health literacy influences the willingness and ability to participate, as a team we spent numerous hours creating and reviewing content to ensure accessibility and using illustration/pictures where possible.

## Conclusions

It is clear from our patient and public engagement exercise that within CYP and their carers a lack of awareness relating to data collection and its use is a leading cause of mistrust within public and patients. Our research groups have implemented these findings into our practice improving the transparency of our research. Listening to the input of CYP and their carers has enabled us to shape our research strategies to ensure our outputs are important and acceptable to those that matter most.

### Supplementary Information

**Additional file 1**. Understanding data for children’s and young people’s cancers - workshop 1 design. Understanding data for children’s and young people’s cancers – workshop 2.

**Additional file 2**. The case studies discussed in workshop 1.

**Additional file 3**. The completed GRIPP2 short form.

## Data Availability

An anonymised version of the datasets used and analysed during the current study are available from the corresponding author on reasonable request.
