# Supplementary material for: Patient and public involvement to inform priorities and practice for research using existing healthcare data for children’s and young people’s cancers
Source: Res Involv Engagem. 2023 Aug 29;9:71. doi: 10.1186/s40900-023-00485-8 (PMC10466824; doi:10.1186/s40900-023-00485-8)
Supplement: Supplementary file 1 — Additional file 1. Understanding data for children’s and young people’s cancers - workshop 1 design. Understanding data for children’s and young people’s cancers – workshop 2. [file 40900_2023_485_MOESM1_ESM.pdf]

Understanding data for children's and young people's cancers - workshop 1 design

Overarching workshop aim: To understand what young people/carers understand about cancer data, how it is collected, what it is used for and how data can improve outcomes through research. Also to identify areas where young people/carers need more information and identify any concerns.

Design of workshop<br>Key: B= In break-out room<br>W= Whole workshop				
Focus group workshop design<br>• We are not merely collecting participants' views, but moving through a process of learning, applying the learning to real-life examples and encouraging debate and reflection to uncover participant-led priorities for future work.<br>• The information we give needs to be unbiased, we should present from all angles and remain neutral in discussions<br>• We need to ensure the well-being and support of participants is paramount. We will have mechanisms for distress, offline support and contact details for the workshop leaders				
Time	Segment	Activities	Mins	Speaker
	Pre-Registration– sent the week before and prompt the day before.	Consent form for audio and photos, instructions on how log in and to change zoom name (Zoom guide).<br>Procedures for support given including contact numbers for any difficulties on the day.<br>Remuneration forms sent in advance with details of how to claim and guidance for time to payment.	n/a	
3:55pm	Zoom Main room opened for welcomes.			
4pm	Introductions and ice breaker (W)	Introduce the process and aims to the whole workshop. Group rules and courtesies.<br><br>Each participant 2 minute introduction.	25	AP/LF
4:30pm	Session 1 – What data is collected about me? (W)	Presentation.	15	CC
5pm	Questions and discussion	Participants are encouraged to ask questions and jot down questions for later on if run out of time or use chat function.	10	NH

5:10pm	Case studies (B)	Group 1 to consider case study 1- Lucy and fill in template (facilitator + professional to go to group)	10	AP/KPJ/CC
		Group 2 to consider case study 2 -Aiden and fill in template (facilitator + professional to go to group)<br><br>N.B Any other workshop leaders to remain in main room to check for any participants that have issues or questions.	10	NH/RF/LF<br><br>EC/AG
5:40	Group discussion about tasks (W)	Each group to nominate a speaker to feedback a summary of discussions. Facilitator to prompt discussion.	10	AP
5:50pm	BREAK	All speakers to go to breakout room to discuss how it is going and any modifications needed	10	
6pm	Session 2 – Why researchers need data? (W)	Group to consider why researchers need data. If they were a researcher what data would they want to have access to and why?	5	AP
6:05pm	Stages of research	Presentation of how the YSRCCYP has used patient data and how it is used at various stages of the research cycle.<br>What do the group think? Facilitator to prompt discussions.	10	NH/RF
6:15pm	Barriers and next steps	Summary of workshop. Participants to write down 3 things they have learnt from today, 3 ways to collect data, 3 possible problems that researchers might have when trying to use patient data.<br><br>Facilitator to and encourage open discussion and discuss potential topics for workshop 2.	15	AP
6:25pm	Thank you and close. Any participants wishing to continue to workshop 2 to send email.		5	AP/CC

<u>Understanding data for children's and young people's cancers – workshop 2</u>

Overarching workshop aim: To build upon the last workshop and discuss issues that participants thought would benefit from a deeper understanding. Also to start to think about outcomes and input from young people, how can they be involved in making a change or increasing awareness. To identify areas where young people/parents need more information and identify any concerns in relation to health data for children and young people with cancer.

Design of workshop<br>Key: B= In break-out room<br>W= Whole workshop				
Focus group workshop design<br>• We are not merely collecting participants' views, but moving through a process of learning, applying the learning to real-life examples and encouraging debate and reflection to uncover participant-led priorities for future work.<br>• The information we give needs to be unbiased, we should present from all angles and remain neutral in discussions<br>• We need to ensure the well-being and support of participants is paramount. We will have mechanisms for distress, offline support and contact details for the workshop leaders				
Time	Segment	Activities	Mins	Speaker
	Pre-Registration– sent the week before and prompt the day before.	Ensure previous consent still viable<br>Procedures for support given including contact numbers for any difficulties on the day<br>Remuneration forms sent in advance with details of how to claim and guidance for time to payment<br>Press clippings and BENCHISTA transparency statement sent for pre-reading	n/a	
3:55pm	Zoom Main room opened for welcomes.			
4:00pm	Introductions and ice breaker (W)	Introduce the process and aims to the whole workshop. Reminder of group rules and courtesies.	10	AP
4:10pm	Questions from last session	Facilitator to give brief summary of what was covered	10	CC
4:20pm	Session 1 – What does cancer health data actually look like?	Presentation of different types of data, when they are collected and by whom. Where are the gaps?	20	CC

4:40pm	Data sharing and data linkage	Brief explanation of the similarities and differences, how would the group explain it in their own words?	10	CC
4:50pm	Project discussion 1 – International data sharing - BENCHISTA		30	AP/KPJ/AL
5:20pm	BREAK	All speakers to go to breakout room to discuss how it is going and any modifications needed.	10	
5.30pm	Project discussion 2 – YSRCCYP – Social outcomes data		30	NH/RF/LF
6:00pm	Trust and communication	Press clippings discussion, what matters to CYP in particular?	25	CC/AP
6.25pm	Next steps	Summary of workshop, run through of ways in which participants can get involved in further projects.	5	CC/EC
6:30pm	Thank you and close. Any participants wishing to volunteer to send email.<br>Briefly outline potential further workshops.		-	CC/EC
