# Supplementary material for: Patient and public involvement to inform priorities and practice for research using existing healthcare data for children’s and young people’s cancers
Source: Res Involv Engagem. 2023 Aug 29;9:71. doi: 10.1186/s40900-023-00485-8 (PMC10466824; doi:10.1186/s40900-023-00485-8)
Supplement: Supplementary file 2 — Additional file 2. The case studies discussed in workshop 1. [file 40900_2023_485_MOESM2_ESM.pdf]

Supplementary material 2. The case studies discussed in workshop 1.

Participants were divided between two breakout rooms. For both cases participants were asked to discuss; what type of data will be collected, who will see it, who will the data be shared with and who needs to consent to sharing the data.

Case A – Lucy

A 9 year old girl who has been diagnosed with Leukaemia. Her Mum and Stepdad have given consent for Lucy to receive treatment. They have also consented for her to take part in a clinical trial. They are happy for Lucy's information to be shared with the hospital and the clinical trial team.

Case B – Aiden

Aiden has been diagnosed with osteosarcoma aged 17 years. He has consented to receive treatment with his parents present. He has also consented to be part of a clinical trial and to have tissue from his tumour stored in the tissue bank.
