# Supplementary material for: Patient and public involvement to inform priorities and practice for research using existing healthcare data for children’s and young people’s cancers
Source: Res Involv Engagem. 2023 Aug 29;9:71. doi: 10.1186/s40900-023-00485-8 (PMC10466824; doi:10.1186/s40900-023-00485-8)
Supplement: Supplementary file 3 — Additional file 3. The completed GRIPP2 short form. [file 40900_2023_485_MOESM3_ESM.pdf]

**Section and topic**	**Item**	**Reported on page No**
1: Aim	Report the aim of PPI in the study	5.
2: Methods	Provide a clear description of the methods used for PPI in the study	5–8 and supplementary files 1 and 2.
3: Study results	Outcomes—Report the results of PPI in the study, including both positive and negative outcomes	8-15.
4: Discussion and conclusions	Outcomes—Comment on the extent to which PPI influenced the study overall. Describe positive and negative effects	15-17.
5: Reflections/critical perspective	Comment critically on the study, reflecting on the things that went well and those that did not, so others can learn from this experience	17.

Supplementary material 3. The completed GRIPP2 short form.
